# A statistical look at the COVID-19 vaccine development and vaccine policies

**DOI:** 10.3389/fpubh.2022.1048062

**Published:** 2022-12-05

**Authors:** Catherine Apio, Kyulhee Han, Gyujin Heo, Taesung Park

**Affiliations:** ^1^Interdisciplinary Program in Bioinformatics, Seoul National University, Seoul, South Korea; ^2^Department of Statistics, Seoul National University, Seoul National University, Seoul, South Korea

**Keywords:** vaccine development, COVID-19, COVID-19 vaccine, SARS-CoV-2, R&D, vaccine policy, liability

## Abstract

The global outbreak of COVID-19 caused by the SARS-CoV-2 virus elicited immense global interest in the development and distribution of safe COVID-19 vaccines by various governments and researchers, capable of stopping the spread of COVID-19 disease. After COVID-19 was declared a global pandemic, several vaccines have been developed for emergency use authorization. The accelerated development of the vaccines was attributed to many factors but mainly by capitalizing on years of research and technology development. Although several countries tried to develop COVID-19 vaccines only a few countries succeeded. Therefore, we applied statistical methods to find factors that have contributed to the fast development of COVID-19 vaccines. All 11 countries that developed vaccines were considered and chose other 24 countries for comparison purposes according to different criteria of their R&D. Fourteen R&D indicator variables that are a measure of the R&D for all countries [World Development Indicators (WDI)] were obtained from the World Bank DataBank and data on the COVID-19 vaccine R&D were obtained from The Knowledge Portal of the Graduate Institute Geneva and Global Health Center. The World Bank records WDI yearly, and 2019 was chosen because of a few missing values. Also, different vaccine policies were adopted by different countries during the COVID-19 vaccination period, producing different impacts of vaccinations on the population. So, we applied the generalized estimating equations (GEE) approach to find policies that contributed greatly to decreasing the spread of COVID-19 using data from the Oxford COVID-19 Government Response Tracker (OxCGRT) and age-specific vaccination data from the European Center for Disease and Prevention and Control. Logistic regression, two-sample *t*-test, and Wilcoxon rank-sum test found scientific and technical journals, liability, and COVID-19 Vaccine R&D Funding (investment in pharmaceutical industry US$) are significantly associated with fast COVID-19 vaccine development. Vaccine prioritization and government vaccine financial support were significantly associated with COVID-19 daily cases. The impact of vaccination on lowering the rate of new cases is greatly observed among the mid-aged populations (25–64 years) and lower or non-significant among the younger (<25 years) and (>65 years) older populations. Therefore, these age-groups especially > 79 can be prioritized during vaccine roll-out.

## Introduction

In the absence of an effective treatment, the outbreak of severe acute respiratory syndrome coronavirus 2 (SARS-CoV-2) causing the coronavirus disease (COVID-19), which became a global pandemic led to unprecedented research to find a cure in the presence of non-pharmacological policies implemented to mitigate and suppress the spread of the virus. SARS-CoV-2 belongs to the Beta coronavirus genus of the Coronaviradae family which is an enveloped single-stranded RNA virus containing a 30 kb genome with 14 open reading frames including four major viral structure proteins: spike (S), membrane (M), envelope (E), and nucleocapsid (N) proteins (1, 2). The genetic sequence of this virus was made public on January 11, 2020, triggering intense global research and development (R&D) activity (3).

Fundamental to all the research was the development of vaccines capable of thwarting the COVID-19 disease, hospitalizations, and associated deaths (4). The Coalition for Epidemic Preparedness Innovations (CEPI) started working with global health authorities, biotech, governments, and academic collaborators to support the development of vaccines against COVID-19 (3, 4). The COVID-19 vaccine R&D landscape developed at an unprecedented scale and speed in that by September 3, 2020, the global COVID-19 vaccine R&D landscape included 321 vaccine candidates (5). This landscape explored a range of technology platforms, not previously employed in currently licensed vaccines but used in different natural science fields. These approaches included nucleic acid (DNA and RNA), virus particles, peptides, viral vectors (replicating and non-replicating), recombinant proteins, live attenuated viruses, and inactivated virus approaches (3). The common types of COVID-19 vaccines that went trails included DNA vaccines, mRNA vaccines, non-replicating non-viral factor vaccines, inactivated vaccines, life attenuated vaccines, and subunit vaccines (6).

The SARS-CoV-2 S-protein became the major target in COVID-19 vaccine development because of its elicitation of neutralizing antibodies during immune response which correlates to vaccine protection (7). Therefore, on December 11, 2020, the U.S. Food and Drug Administration issued the first emergency use authorization (EUA) for the Pfizer-BioNTech COVID-19 vaccine for the prevention of COVID-19 in individuals 16 years of age and older (8, 9). After that, other countries followed and issued approval including the Moderna vaccine, Oxford-AstraZeneca vaccine, Sputnik V vaccine, and Johnson & Johnson vaccine (10). These vaccines are mainly composed of the S-antigen and exist either as inactivated vaccines, non-replicating viral vector vaccines, subunit vaccines, or RNA vaccines.

These capitalized on years of progress on new vaccine platforms, viral immunology, structure-based antigen design, computational biology, protein engineering, and gene synthesis, along with clinical trial operations expertise provided tools to enable the rapid development, evaluation, manufacturing, and deployment of successful vaccines (4, 11). So, many factors have been attributed to the accelerated development and deployment of COVID-19 vaccines including years of research, new technology, funding, collaborations and partnerships, fast clinical trials, reduced regulations, rapid response to outbreaks, and so forth (12, 13). Given this unexpected outbreak of the SARS-CoV-2 pandemic, governments and researchers are confronted with the issue of being able to prevent another outbreak from becoming a global pandemic in case of a virus of this or any kind.

The fast development of a good vaccine is expected to play a game changer. Therefore, it is paramount to find out the factors most important to the successful development of vaccines, given that many countries tried to develop COVID-19 vaccines but only a few succeeded. In addition, different vaccine policies were adopted by different countries during the COVID-19 vaccination period, producing different impacts of vaccinations on the population. So, the policies that bring the maximum impact of vaccination need to be known. Here, statistical methods were employed to (3) find factors that may have played a role in the fast development of COVID-19 vaccines using real data from the World Bank and COVID-19 R&D indicators (14, 15) and (4) the impact of vaccination and vaccine policies on COVID-19 confirmed cases (if these vaccines worked in lowering the spread of COVID-19) using OxCGRT data. This study considered 35 countries of which 11 developed at least one COVID-19 vaccine and 24 countries that did not develop a vaccine were selected for comparison purposes. Also, specific age-group vaccine population data and COVID-19 cases were analyzed.

## Materials and methods

### R&D indicator variables' data

Firstly, the list of countries and approved COVID-19 vaccines used in the mass vaccination of the world population was obtained online from (16, 17). Fourteen variables called the World Development Indicators (WDI) which are the R&D indicators for all countries for the latest year 2019 were downloaded from the World Bank DataBank (15). For a country with a missing data point, the value of the last recorded year was used. The COVID-19 R&D data that records the different sources of funding and the recipients of the funds concerning COVID-19 vaccine development was obtained from The Knowledge Portal of the Graduate Institute Geneva and the Global Health Center website (15). This data was the source for variables like government funding, non-government funding, total funding, investment in the pharmaceutical industry, and investment in academic or research institutions. Liability was recorded as a binary variable if a given country had a COVID-19 vaccine liability agreement with the source of the vaccine.

We considered all 11 countries which have developed a vaccine. For comparison purposes, we selected other 24 countries with potential for vaccine development due to their high R&D indices as listed in these articles (18–22). The response variable was binary, 1 whether a country developed a vaccine and 0 otherwise. The list of the countries is in Table 1 above and the R&D indicator variables are described in Table 2 below.

**Table 1:** List of countries selected, and vaccines developed.

**Vaccine development**	
**Developed vaccine (11 countries)**	**Non-developed vaccine (24 countries)**
Australia (COVAX-19), China Mainland (SARS-CoV-2, COVID-19 Inactivated, BBIBP-CorV, Inactivated (Vero Cells), CoronaVac, Ad5-nCoV, ZF2001), Germany (BNT162b2), India (ZyCoV-D, Covishield, COVOVAX), Iran (COVAX-19), Kazakhstan (QazVac), Russia (KoviVac, Sputnik Light), Sweden (AZD1222), United Kingdom (AZD1222), United States (mRNA-1273, BNT162b2), Cuba (CIGB-66)	Argentina, Austria, Belgium, Brazil, Canada, Denmark, Finland, France, China, Hong Kong, Indonesia, Israel, Italy, South Korea, Malaysia, Mexico, Netherlands, Norway, Portugal, Singapore, South Africa, Spain, Switzerland, Ireland, Japan

**Table 2:** List of the R&D indicator variables.

**Variable**	**Explanation**
**World bank data**	
High-technology exports (% of manufactured exports)	High-tech products export ratio
High-technology exports (current US$)	Exports of high-tech products
Technicians in R&D (per million people)	Number of technicians involved in R&D
Researchers in R&D (per million people)	Number of researchers engaged in R&D
Trademark applications, total	Number of trademark applications
Trademark applications, direct resident	Number of trademark applications (if a resident applies directly)
Trademark applications, direct non-resident	Number of trademark applications (if a non-resident applies directly)
Patent applications, residents	Number of patent applications (resident applications)
Patent applications, non-residents	Number of patent applications (non-resident application)
Scientific and technical journal articles	Number of scientific and engineering papers
Research and development expenditure (% of GDP)	R&D expenditure as a percentage of the GDP
Charges for the use of intellectual property, receipts (BoP, current US$)	Intellectual property income
Charges for the use of intellectual property, payments (BoP, current US$)	Intellectual property usage fee
Income Group (developed/non-developed)	Lower middle (2/1), Upper middle (4/5), High (6/17)
**COVID-19 vaccine R&D data**	
Non-government funding (US$)	If the funding is from another country, private entity, or public contributions
Government funding (US$)	If the recipient country's government is the source of funds
Funding, total (US$)	Sum of government and non-government funding
Investment in the pharmaceutical industry (US$)	If the funding went directly to a pharmaceutical company
Investment in academics or research institutes (US$)	If a university or research institute is the direct recipient
Liability (developed/non-developed)	A damage exception agreement exists between the country and the vaccine company. Yes (5/19), No (5/1), and Unknown (2/3)

### OxCGRT vaccine policy data

Secondly, three vaccine policies (Table 3); vaccine prioritization, vaccine eligibility, and vaccine financial support obtained from the OxCGRT dataset (23), were analyzed for their impact on COVID-19 daily cases. Since these policies were recorded on an ordinal scale, each policy was divided by its maximum value and multiplied by 100 thus ranging from 0 to 100. Data smoothing using a simple moving average (SMA) with window size 7 was applied to the policy data including the daily confirmed cases, to remove the periodicity found in the data, and missing or negative values were replaced with zero. The same 35 countries were analyzed, and the period was from January 1st, 2021 to May 31st, 2022, just before the rapid spread of the omicron variant.

**Table 3:** COVID-19 vaccine policies from the OxCGRT data.

**OxCGRT vaccine policy data**	
Vaccine prioritization	Vaccination priority by age (in 5-year units), risk, and occupation (medical occupation, military, etc.)
Vaccine eligibility	Vaccination availability by age (5 years unit), risk, and occupation (medical occupation, military, etc.)
Vaccine financial support	Financial support for vaccination by age (in 5-year units), risk, and occupation (medical occupation, military, etc.)

### Age-group specific data

The impact of vaccination on the spread of COVID-19 is usually observed by analyzing the relationship between COVID-19 cases and the rate of vaccination or proportion of the population vaccinated. However, if the COVID-19 cases and vaccination data are divided into specific age-groups, the impact of vaccination per age-group can be observed and this informs which age-group should be prioritized for maximum control of the spread of COVID-19 disease. So, six age-groups in years were considered namely <15, 15–24, 25–49, 50–64, 65–79, and 80+ years. The data recording share of the population vaccinated by age groups was obtained from (24). This data reports vaccine doses per age-group, year-week, population per age-group, and reporting country. The second dose recording per age-group was considered in our analysis. The age-specific rate of new cases' data was obtained from (25). This data records the rate of newly reported COVID-19 cases per one thousand of the population by age group, year-week, and reporting country. Both data are weekly which were converted to represent per hundred thousand of the population, and the year-weak of 2021 was selected as the analysis period. The impact of vaccination on the rate of new cases was the goal of this analysis. Only 30 countries out of the 35 countries were available in the datasets and so analyzed. Of the 30 countries, Germany provided age-group information for <60 and 60+ age-groups only. Liechtenstein and Netherlands provided only total population information. Assuming the same rate of vaccination for all age-groups, for Liechtenstein and Netherlands, the total information was used for all age-groups while for Germany, <60 information was used for all age-groups below 60, and 60+ information was used for above 60 age-group.

### Statistical analysis

Common statistical methods like logistic regression, two-sample *t*-test, Fisher's exact test, K-means clustering, and principal component analysis (PCA) were applied to compare the vaccine-developed and non-vaccine developed groups of countries using the above-mentioned R&D indicator variables. Continuous variables were standardized to have a mean zero and a standard deviation of one before logistic regression analysis.

For the vaccine policies and the age-group-specific data, the generalized estimation equation (GEE) approach (26, 27) was applied to analyze these two datasets. The model is defined as below:


(1) $$\log(\mu_{i}(t))=\beta_{0}+\beta_{1}t+\beta_{2}\log(t)+\beta_{3}Z_{i}(t-lag)$$

where μ_*i*_ = *E*(*Y*_*i*_), *Y*_*i*_(*t*) are the daily COVID-19 confirmed cases or specific age-group rate of new cases, *t* is the number of days or weeks since the first case, β0, β1 and β_3_ are the regression coefficients, *Z*_*i*_(*t*)s are the different vaccine policies or specific age-group vaccination, for each country *i*. The Poisson distribution and the log link function were used. An independent working correlation was assumed. 0, 2 weeks, 1, 2, and 3 months lagging (*lag*) were assumed when analyzing the vaccine policies' data but not in the age-group specific analysis because it is bi-weekly and not daily data. A lag is a fixed time displacement in time series data. This assumes that the effects of policies implemented on a given day may affect the number of confirmed cases several days after implementation. Statistical significance level was taken for *p*-values (*P*) < 0.05. All countries and cluster analyses using groups of countries produced from the K-means clustering analysis were performed for both vaccine policy analysis and specific age-group analysis. All analyses were carried out in the R (ver. 4.1.0) software tool.

## Results

### Vaccine development analysis

**Figure 1:**
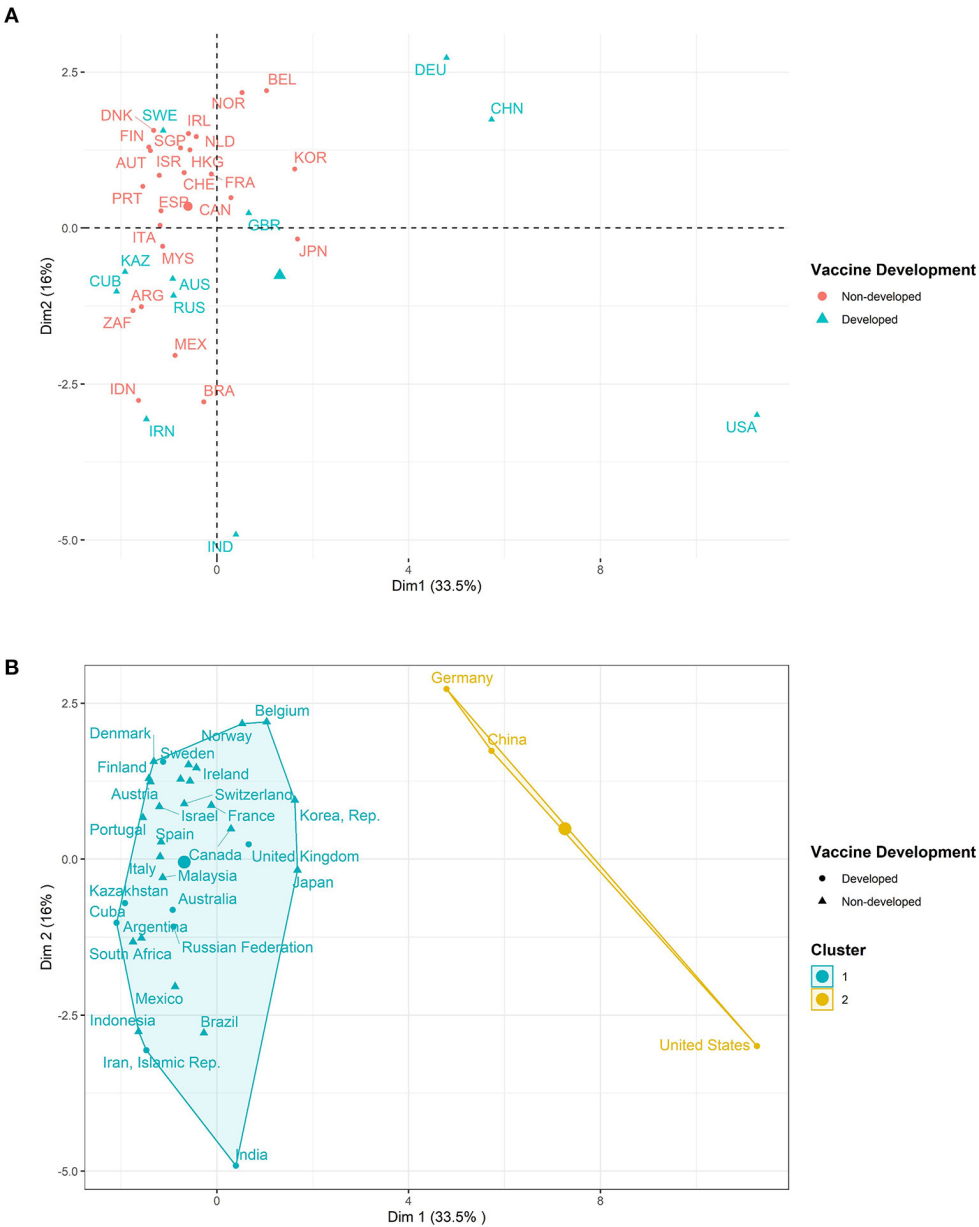
Unsupervised clustering methods. **(A)** Principal component analysis. **(B)** K-means clustering.

In total, 20 variables were analyzed for their association with fast COVID-19 vaccine development. Exploratory analysis using unsupervised clustering methods of PCA and K-means clustering did not reveal noticeable differences between the two groups of countries. Figure 1 shows the results of PCA and K-means clustering. K-means (k = 2) classified the countries into two groups with one cluster having the USA, China, and Germany and the other cluster having the remaining 32 countries. K-means misclassified 8 countries belonging to the vaccine-developed group as belonging to the non-developed vaccine group. The same could be observed with PCA as it could not differentiate between the two groups of countries. Both methods captured 33.5 % and 16% variance of the data in the 1st and 2nd dimensions, respectively.

**Figure 2:**
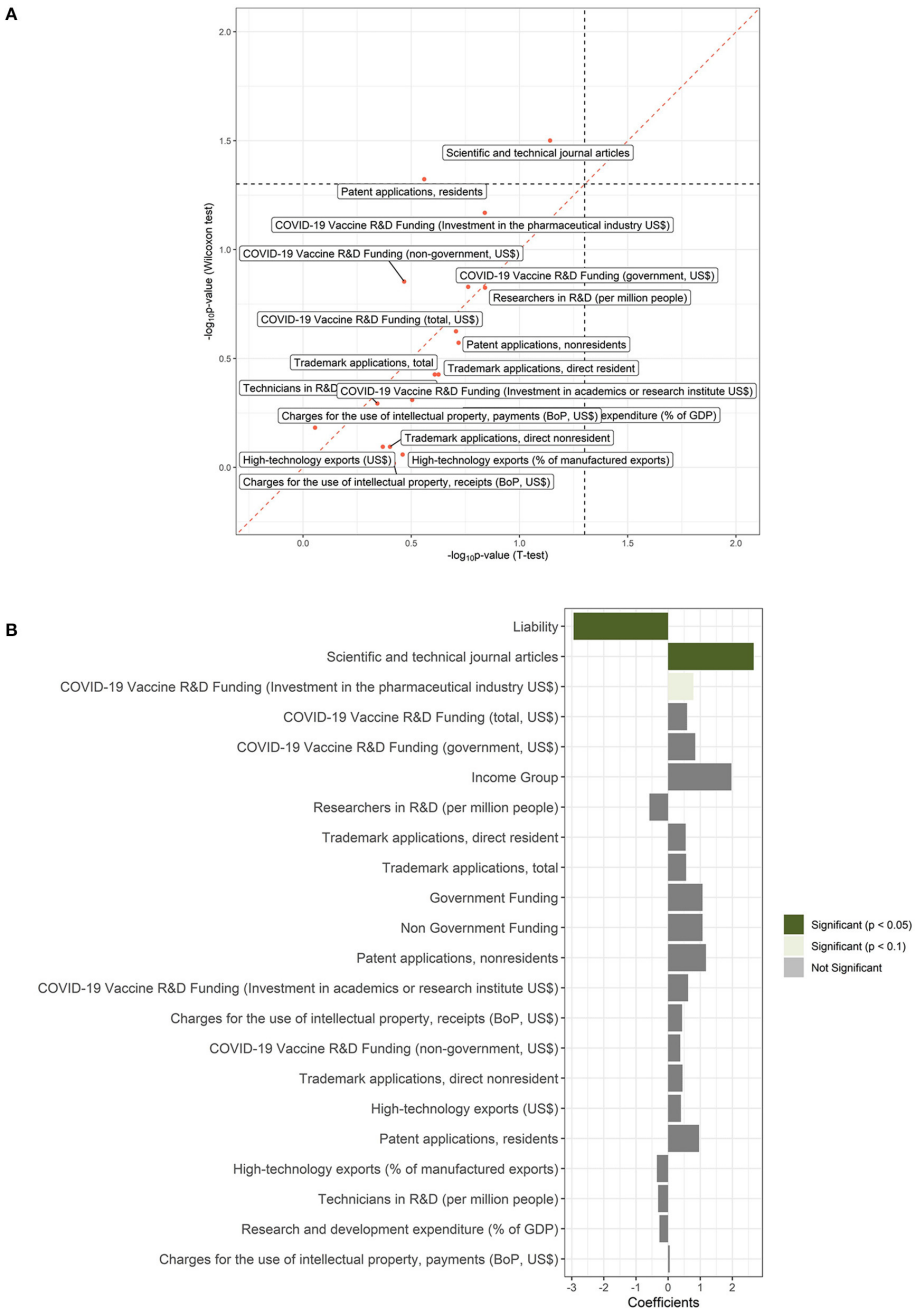
Results of association analysis. **(A)** -log10 (*P-*value) values of the continuous variables of the *t*-test vs. the Wilcoxon test. **(B)** Results from the simple logistic regression. The bars show the effect sizes and the colors show the level of significance by *P*-values. Significance means *P* < 0.05, Evidence means *P* < 0.1 and not-significance means *P* > 0.1.

For numeric variables, a two-sample *t*-test and the non-parametric Wilcoxon rank-sum test were applied (Figure 2A). *T*-test found all R&D indicator variables except scientific and technical journal articles to be non-significantly associated with fast vaccine development. However, Wilcoxon rank-sum test found all R&D indicator variables to be not significant except patent applications (residents), COVID-19 Vaccine R&D Funding (investment in the pharmaceutical industry US$), and scientific and technical journal articles. Fisher's exact test found liability to be significantly (*P* = 0.0088, odd ratio = 0.05982) associated with fast vaccine development, and the income group was not significant. Furthermore, logistic regression found scientific and technical journal articles, liability, and COVID-19 Vaccine R&D Funding (investment in the pharmaceutical industry US$) to be associated with vaccine development (Figure 2B).

### Vaccine policy and age-group analysis

**Figure 3:**
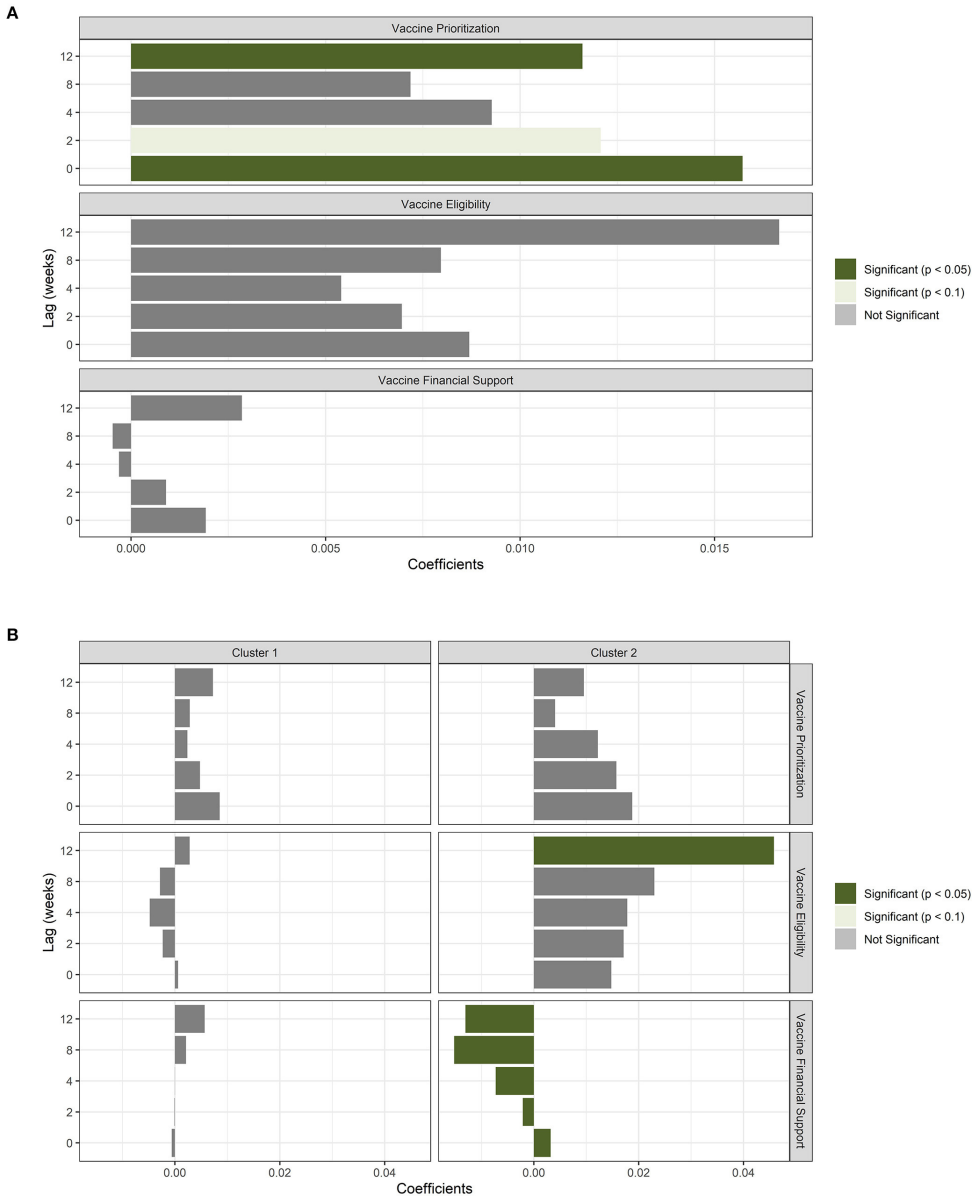
Vaccine policy results in lagging. **(A)** Results from the analysis of vaccine policies using all countries. **(B)** Results from the analysis of vaccine policies for cluster groups of countries.

**Figure 4:**
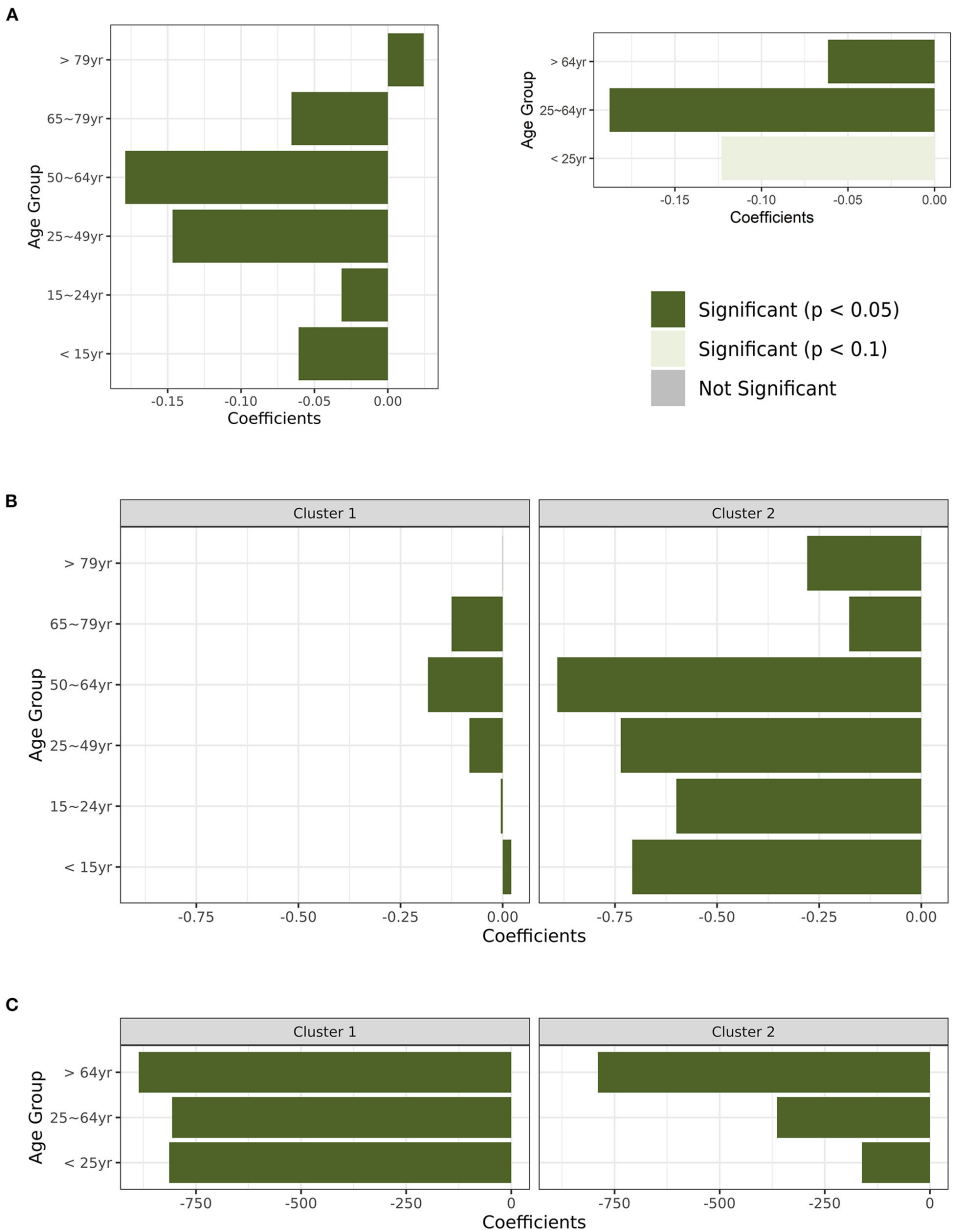
Results for specific age-group analysis of vaccination on the rate of new COVID-19 cases. **(A)** All countries with six age-groups and three age-groups. **(B)** Cluster groups with six age-groups. **(C)** Cluster groups with three age-groups.

Analysis of the three vaccine policies using the GEE approach found only vaccine prioritization to be significant with no lagging and at 12 weeks lag, albeit not a negative relationship (Figure 3A). However, the grouping of the population vaccination rate and the confirmed cases into specific age-groups revealed the impact of vaccination in lowering the rate of new confirmed cases for all age-groups except the >79 years age-group, especially among the population aged 25–49 and 50–64 years (Figure 4A). For the age-group >79 years, though the relationship between vaccination and the rate of new cases is significant, vaccination does not have a lowering effect on the rate of COVID-19 cases. The effect size is small (0.025) and the relationship with vaccination is positive. For the combined age-groups, <25 years does not have a significant relationship between vaccination and the rate of new cases. But, we observe the > 65 years age-group shows a lowering impact of vaccination on the rate of new COVID-19 confirmed cases (Figure 4A).

K-means clustering analysis clustered the countries into two clusters with cluster 1 having 32 countries and cluster 2 having three countries (China, USA, and Germany). Contrasting the three vaccine policies between the two clusters found no policy significant in the Cluster 1 countries. But, cluster 2 countries' analysis found vaccine eligibility at the 12 weeks lag and vaccine financial support at all lag points to be significantly and negatively associated with COVID-19 daily confirmed cases (Figure 3B). For specific age-group analysis, age-group data was provided for only European countries with 12 out of 30 countries belonging to cluster 1 and 1 country (Germany) to cluster 2. The two clusters yielded similar results of the significantly lowering impact of vaccination on the rate of new COVID-19 cases (Figures 4B,C).

## Discussion

Due to the unprecedented development and roll-out of COVID-19 vaccines, we looked at some factors that may have contributed to this outcome. Twenty variables (Table 2) related to R&D and funding from the World Bank database and the compiled COVID-19 R&D investment for 35 countries were analyzed using simple statistical methods like the parametric two-sample *t*-test, non-parametric Wilcoxon rank-sum test, Fisher's exact test, and logistic regression. The outcome was binary whether a country developed a vaccine (11 countries) or not (24 countries).

Firstly, unsupervised learning methods like PCA and K-means clustering applied to find the existence of patterns in the data revealed no distinct variable patterns between the two groups of countries except for the countries of the USA, China, and Germany (cluster 1). Secondly, Fisher's exact test revealed the significant association of liability with vaccine development. Scientific and technical journal articles, liability, and COVID-19 Vaccine R&D Funding (investment in pharmaceutical industry US$) were significantly associated with accelerated vaccine development using logistic and Wilcoxon-rank sum test. A key element in the accelerated development of COVID-19 vaccines was attributed to the ability to apply the extensive vaccine-development experience of industry and academia (12). Journal articles record the different research, new developments, and technologies happening in the academic and industrial fields. Therefore, they can be considered as the unit of R&D for a given country in the different fields of natural science and technology. This reveals the great contribution of years of research, especially in the areas of immunology, structural biology, protein engineering, high throughput sequencing, and mRNA research in the development of COVID-19 vaccines. All knowledge and research required for the successful COVID-19 landscape were shared through these journal articles. Liability shortens the period from vaccine conception to roll-out since the time required for clinical trials and side-effects study is greatly reduced. Investment in the pharmaceutical industry especially government investment further increased the efforts put into the development of a given vaccine. Much more effort is placed into crucial R&D for long-term new drugs or vaccines. Clinical trials are very expensive thus making them take a long time but investments can shorten the time of clinical trials making the vaccines more available.

All these factors are measures of R&D indicators of a given country. The evidence of the importance of R&D especially in new technology and investment in the pharmaceutical industry can be observed. The crucial roles of funding especially government funding and liability cannot be ignored. For emergency use during a health crisis, liability may be considered, however, for non-emergency use, the safety and liability process surrounding a future vaccine becomes prominent (28, 29). Technology-based and R&D factors are the two main factors identified concerning the rapid COVID-19 vaccine development in literature. Our results statistically provide evidence for the roles of these factors in vaccine development. Because of the difference in the number of countries in the vaccine-developed (11 countries) and non-vaccine developed groups (24 countries), it is slightly less powerful when comparing these two groups as some might argue, especially if a class imbalance exists between the two classes. However, there is no bias in our estimations for the two groups since we think such a minor difference does not favor the variables being assessed.

Because the demand for COVID-19 vaccines exceeded the supply, phased distribution and prioritization for first responders, essential workers, older adults and high-risk medical condition individuals (30) was the basis of most governments during vaccine allocation to its citizens. The goal was to save lives at risk and reduce the spread of COVID-19 as much as possible. Vaccine prioritization was significantly associated with daily COVID-19 confirmed cases. However, its impact was not negative as assumed before the analysis. This can be attributed to its impact being not strong enough to cause an immediately noticeable decrease in daily confirmed COVID-19 cases. Also, since the GEE approach provides marginal results (irrespective of other factors), the conditional analysis may be required to control for other unknown factors not considered that affect spread of COVID-19. In addition, vaccine prioritization may have been too late to have an impact given that the disease was already widespread across the globe when vaccine roll-out was commenced.

Group analysis between cluster 1 and cluster 2 countries from the K-Means analysis revealed the importance of vaccine eligibility and the negative impact of vaccine financial support for cluster 2 countries in decreasing the spread of COVID-19. Cluster 2 has the USA, China, and Germany. Vaccine eligibility recorded the categories of people receiving vaccines regardless of their position in the prioritized roll-out plan. Since most countries including cluster 2 opened vaccination early to all their citizens except children and babies, we observe the importance of making vaccines available to everyone playing an important role in trying to control the spread of COVID-19. Therefore, prioritization and eligibility can be combined to give a more effective vaccine policy. Vaccine financial support recorded how vaccines were being funded for each category of people identified eligible in vaccine eligibility. This included costs borne by the individual or private health insurance, partially government-funded and fully covered by the government. COVID-19 vaccine costs were fully covered by governments during the height of the pandemic making it readily available to all. So, when a pandemic breaks out and vaccines are the only hope in reducing the number of cases and severity, governments must make them readily available to the public to control the pandemic as observed in this analysis. Also, cluster 2 countries roll out vaccinations way earlier than most countries showing the importance of fast and readily available treatment during a pandemic.

But, looking at the impact of vaccination on the rate of new cases in different age groups revealed the negative impact of vaccination in reducing COVID-19 cases. This impact is greatly observed among the mid-aged populations (25–64 years) and lower (or non-significant) in younger (<25 years) and older (>65 years) populations. The most impacted age group was the 25–49 years age-group and the 50–64 years age-group. Aggregating the six age groups into three age groups (<25, 25–64, 65+) revealed the lowering but diminishing impact of vaccination with increasing age (Figure 4A). Group analysis of cluster 1 vs. cluster 2 countries showed the lowering impact of vaccination on the rate of new cases for all age-groups except <15 in cluster 1 which has a positive relationship (Figure 4B). Group analysis results are not conversely different from the analysis that uses all countries except that the impact of vaccination on the rate of new cases for the >64 years age-group relationship becomes negative and more profound (Figure 4C). Since the goal of prioritization was the target of risk groups, the elderly are the right group to be targeted first from the results. However, if the goal is where the effect of vaccination will be highly observed, then the younger and mid-aged populations should be targeted. During the pandemic, this group (<25) has been the greatest carriers of the virus within the population and passing it on to the elderly members of the family, owing to them having asymptomatic characteristics when infected with the SARS-CoV-2 virus and a high level of mobility. Others can argue that with them protected, then the older population will be indirectly protected. However, the effect of other government-implemented policies per age-group is required to make a more concise decision.

Our analysis had some limitations. A histogram of the variables revealed that the data was right-skewed with many outliers. Standardization removed the skewness but not the outliers. Logistic regression of these R&D indicator variables outputs most variables with large standard error values despite large coefficient values, thus making most variables non-significant, being worsened by the small sample size. This phenomenon persisted with log-transformed and standardized data. In addition, we want to consider the efficacy of the vaccine depending on the type of vaccine and the proportion of SARS-CoV-2 variants in future analyses.

In conclusion, except for scientific and technical journal articles, other factors that played a role in fast COVID-19 vaccination were not significantly observed but evidence of the roles of these factors especially technology, research, liability, and mass funding was noticed throughout the analysis. This may be attributed to our small sample size which reveals that a more in-depth analysis with a larger sample size is required. Also, in the next vaccine roll-out either due to a newer outbreak of a disease, population risk, or/and government policy, especially in cases when vaccination does not completely stop the spread of the disease like in the COVID-19 disease, then age-group stratification of the population is important to achieve maximum impact of the vaccines.

## Data availability statement

The original contributions presented in the study are included in the article/supplementary material, further inquiries can be directed to the corresponding author.

## Author contributions

TP: conceptualization, supervision, project administration, and funding acquisition. TP and CA: methodology. CA, KH, and GH: formal analysis. TP and KH: resources. CA: writing—original draft preparation. CA, TP, and KH: writing—review and editing. All authors contributed to the article and approved the submitted version.

## Funding

This research was supported by research grants from the Ministry of Science and ICT, South Korea (No. 2021M3E5E3081425).

## Conflict of interest

The authors declare that the research was conducted in the absence of any commercial or financial relationships that could be construed as a potential conflict of interest.

## Publisher's note

All claims expressed in this article are solely those of the authors and do not necessarily represent those of their affiliated organizations, or those of the publisher, the editors and the reviewers. Any product that may be evaluated in this article, or claim that may be made by its manufacturer, is not guaranteed or endorsed by the publisher.
